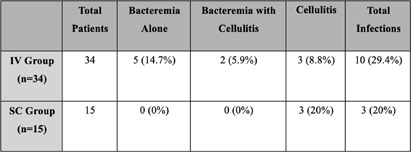# Comparison of Infection Risks Between Intravenous and Subcutaneous Treprostinil in Patients with Pulmonary Hypertension

**DOI:** 10.1017/ash.2025.346

**Published:** 2025-09-24

**Authors:** Maria Akiki, Chebly Dagher, Kristen Swanson, Brett Carollo, Raj Parikh

**Affiliations:** 1 University of Connecticut; 2 Hartford Healthcare; 3 Hartford Hospital;.

## Abstract

**Background:** Treprostinil, a prostacyclin analog, is used to manage pulmonary hypertension (PH) through continuous intravenous (IV) infusion via a central venous catheter (CVC) or continuous subcutaneous (SC) infusion via a small infusion pump connected to a catheter. This study compares the incidence and the types of infections between IV and SC administration in a single-center cohort. **Methods:** We analyzed 49 PAH patients receiving treprostinil at the Hartford Hospital PH Center, all managed under standardized hygiene protocols by the same healthcare team. Of these, 34 received IV administration and 15 SC, based on patient preference and PH specialist recommendations. The primary outcome was the incidence of infection in each group during the study period. The secondary outcome was the type of infection, including bacteremia, cellulitis, or other skin infections, associated with IV or SC administration. **Results:** The incidence of bacteremia was significantly higher in the IV group, with 7 cases (5 isolated bacteremia and 2 bacteremia with cellulitis), representing 20.6%. In contrast, there were no bacteremia cases in the SC group. Cellulitis was more common in the SC group (20%; 3 out of 15 patients) compared to the IV group (8.8%; 3 out of 34 patients). Notably, 2 cases of cellulitis in the IV group were associated with bacteremia, while all 3 cases in the SC group were isolated, with 1 progressing to an abscess requiring incision and drainage. The overall infection rate (bacteremia and cellulitis combined) was higher in the IV group (29.4%) compared to the SC group (20%). These findings emphasize the higher risk of bacteremia in the IV group and reveal that while cellulitis occurred more frequently in the SC group, the overall infection burden was greater in the IV group. **Conclusion:** Previous studies show comparable efficacy between IV and SC remodulin when properly dosed. Our findings, despite a small sample size, reveal a higher overall risk of infections, particularly bloodstream infections (BSIs), with IV therapy due to CVC use. This aligns with existing literature identifying catheter-related infections as a key concern. These results support SC remodulin as a safer option, especially for reducing BSI risk. We plan to incorporate these findings into our counseling protocol, acknowledging the need for further validation.

**Figure:**